# Identify Inflammatory Bowel Disease-Related Genes Based on Machine Learning

**DOI:** 10.3389/fcell.2021.722410

**Published:** 2021-07-26

**Authors:** Lili Ye, Yongwei Lin, Xing-di Fan, Yaoming Chen, Zengli Deng, Qian Yang, Xiaotian Lei, Jizong Mao, Chunhui Cui

**Affiliations:** ^1^Daycare Chemotherapy Center, Zhujiang Hospital, Southern Medical University, Guangzhou, China; ^2^Department of General Surgery, Zhujiang Hospital, Southern Medical University, Guangzhou, China

**Keywords:** index term-inflammatory bowel disease, IBD-related genes, SVM, machine learning, disease ontology

## Abstract

The patients of Inflammatory bowel disease (IBD) are increasing worldwide. IBD has the characteristics of recurring and difficult to cure, and it is also one of the high-risk factors for colorectal cancer (CRC). The occurrence of IBD is closely related to genetic factors, which prompted us to identify IBD-related genes. Based on the hypothesis that similar diseases are related to similar genes, we purposed a SVM-based method to identify IBD-related genes by disease similarities and gene interactions. One hundred thirty-five diseases which have similarities with IBD and their related genes were obtained. These genes are considered as the candidates of IBD-related genes. We extracted features of each gene and implemented SVM to identify the probability that it is related to IBD. Ten-cross validation was applied to verify the effectiveness of our method. The AUC is 0.93 and AUPR is 0.97, which are the best among four methods. We prioritized the candidate genes and did case studies on top five genes.

## Introduction

Inflammatory bowel disease (IBD) (Graham and Xavier, 2020) is a worldwide high incidence of intestinal inflammation, which is divided into Crohn’s disease (CD) (Roda et al., 2020) and ulcerative colitis (UC) (Danese et al., 2020). It can lead to diarrhea, rectal bleeding, abdominal pain, and malnutrition. In addition, IBD is a major risk factor for colorectal cancer (CRC) (Cheng et al., 2020; Olén et al., 2020). The incidence rate of colon cancer in IBD patients is 18 times than that of the general population. In view of the increasing incidence rate of IBD worldwide and the huge medical burden, IBD has become a public health problem which needs to be solved urgently. Therefore, it is of great significance to carry out the research on the pathogenesis of IBD (Mayer, 2010), which enables the development of IBD drug targets for the prevention and treatment.

At present, it has been found that the main causes of IBD are the destruction of intestinal microorganism homeostasis, the lack of intestinal epithelial barrier function and the disorder of innate/adaptive immune system, which are caused by the external environmental factors such as smoking, obesity, eating habits (Hovde et al., 2014). Studies also reported that genetic factors such as MUC2 (Van der Sluis et al., 2006) and IL10 (Franke et al., 2008), also play an important role in the development of IBD. In view of the important role of gene mutation in the development of IBD, the genome wide association studies (GWAS) have been applied to the risk prediction and mechanism of IBD (Franke et al., 2008; Zhang et al., 2021). In addition to the discovered NOD2, IL-23R, ATG16L1, IL-10R, IL-10, XIAP, etc. of IBD-related genes in European and American populations, recent studies have reported more new involvement in cell autophagy, immune regulation, intestinal mucosal barrier, etc. The total number of functional IBD-related sites has reached 200. However, GWAS can only identify the relationship between one single mutation and IBD. The biological functions of mutation and the significance of each gene cannot be explored by GWAS. Although many research have combined expression quantitative trait loci (eQTL) with GWAS to explore the biological functions, this method cannot perform large-scale disease-related gene prediction (Zhao et al., 2019, 2020c).

With the development of computational methods, the applications of algorithms have widely changed the way of doing biological research (Tianyi et al., 2020; Zhao et al., 2020a). Computational methods such as machine learning and deep learning have achieved great achievement in discovering biological mechanism (Zhao et al., 2020b). Predictions by algorithms have shown power in identifying multiple aspects of diseases, such as genes, RNAs, proteins, metabolites, drugs, drug targets, etc. (Zhao et al., 2021). The most common hypothesis of these computational methods is similar diseases are related to similar genes or similar genes cause similar diseases. Research have reported that many disease susceptibility genes do not exist independently, but are associated with a variety of diseases, including IBD. The latest research found that the n2081d allele of LRRK2 gene, which is closely related to the risk of CD, is located in the kinase domain of G2019S gene (Poulopoulos et al., 2012), and G2019S is one of the major mutations involved in familial or sporadic Parkinson’s disease. It is also found that three variants of SIAP (s123n, r233q, p257a) of the primary immune deficiency disease type 2 X-linked lymphoproliferative disease related mutation genes (s123n, r233q, p257a) are related to the activation of CDsusceptible gene NOD2 signaling pathway, which is closely related to CD pathogenesis. Therefore, it is theoretically supported to identify IBD related genes from IBD similar diseases.

In this paper, we purposed a Support Vector Machine (SVM)-based on method to identify IBD-related genes. Based on all known genes related to IBD and known related genes of diseases similar to IBD, we constructed a gene network by gene interactions. The features of each gene were extracted from this network and inputted into SVM to identify the pattern that genes are related to IBD.

## Materials and Methods

### Diseases Similar to IBD

Semantic-based methods use the correlation between ontology and the number of disease-related genes to calculate disease similarity. Obviously, not all associations between diseases are represented by ontology, and some of them are reflected by functional associations between disease-related genes. Semfunsim (Cheng et al., 2014) uses disease-related gene sets in a weighted network of human gene functions to calculate disease similarity.

**FIGURE 1:**
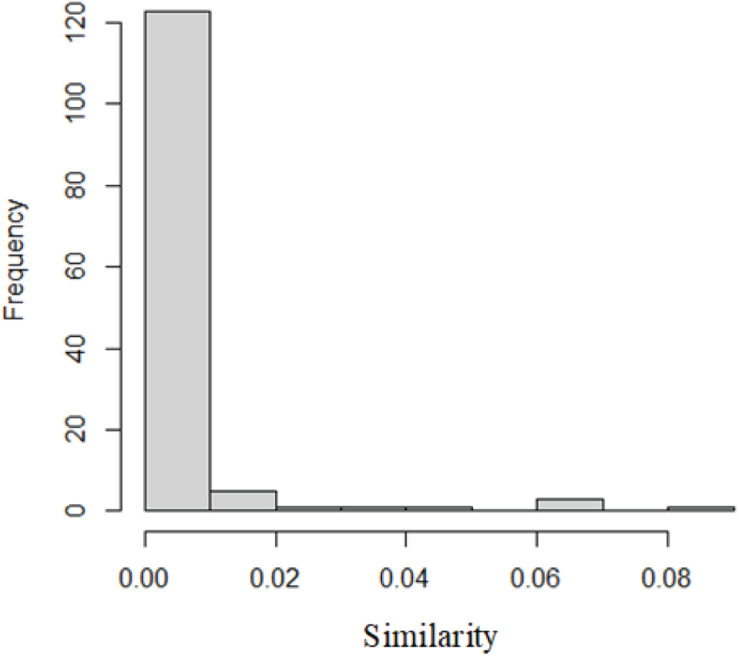
The distribution of similarities.

In this paper, we used the results of Semfunsim. One hundred thirty-five diseases have similarities with IBD according to the results of Semfunsim. The distribution of similarities is as Figure 1.

The disease most similar to IBD is lower respiratory tract disease with similarity of 0.08. The second one is sensory system disease.

### Candidate Genes

We obtained disease-related genes from DisGeNET (Piñero et al., 2016) which collects experimentally verified disease-related genes. Many of the 135 diseases are subtypes of diseases, so their related genes cannot be found in DisGeNET.

**FIGURE 2:**
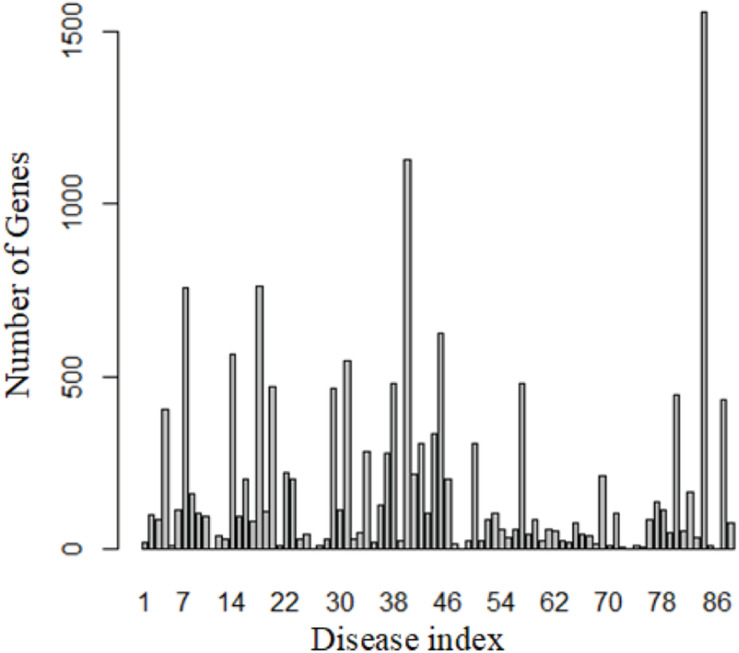
Number of known genes for each disease.

We totally obtained 5,928 genes from 88 diseases by DisGeNET. Figure 2 shows the distribution of these genes.

These 88 diseases have 15,271 entries, which means 15,271 genes are known related to these 88 diseases. However, most of these genes related to more than one disease, so only 5,928 unique genes were obtained. This also reveals that similar diseases share similar genes.

### Workflow of SVM-Based Method

We obtained gene interactions from HumanNet v2.0 (Hwang et al., 2019) and constructed a gene interaction network based on candidate gene interactions.

**FIGURE 3:**
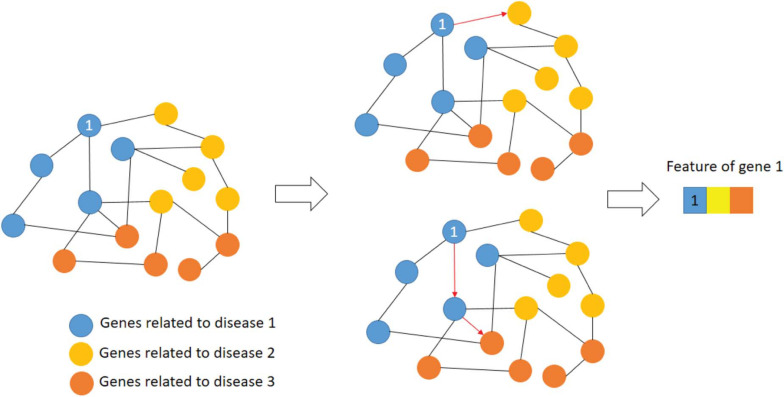
Example of gene feature extraction.

The features of a gene are expressed as the shortest path to other disease-related genes. Figure 3 shows an example of extracting gene features.

As shown in Figure 3, the blue nodes represent the genes related to disease 1, yellow nodes for disease 2 and orange nodes for disease 3. The features of first blue gene could be represented as the shortest paths to disease 2 and 3. The edge of this network is the interaction strength. Therefore, the first dimension of gene 1 is 1 since this gene is related to disease 1 directly. The second dimension of gene 1 is the interaction strength between gene 1 and the yellow node.

If the network is huge, we cannot extract features of genes manually. Here, we applied Graph search Dijkstra algorithm (Noto and Sato, 2000) to search the shortest path. The algorithm gradually diffuses outwards with the starting point as the center, and the shortest path can be obtained.

Assuming that each node in the network has a label (*d*_*t*_,*p*_*t*_),*d*_*t*_ is the shortest path length from the starting point *s*to the point *t*, and *p*_*t*_ represents the point before the midpoint of the shortest path from*s*to the point *t*. If we want to find the shortest path, we need to initialize the starting point.

(1) $$\begin{array}{c} d_{k}=0\\ d_{i}=\infty \end{array}$$

K is the starting point and *p*_*k*_ is NULL.

Then, verify the distance from all marked points *k* to other directly connected unmarked points *j*, and set:

(2) $$d_{j}=\min\left[d_{j},d_{k}+w\,\left(k,j\right)\right]$$

*w*(*k*,*j*) represents the length from *k* to *j*.

Then, pick the next point. The smallest point *i* is selected from all unmarked points *d*_*i*_, and the point *i* is selected as the point in the shortest path. Find the point directly connected to the point from the set of marked points, and mark it as *p*_*i*_. Mark the point *i*. If all the points are marked, the algorithm ends. The flow of this algorithm is as Table 1.

**TABLE 1:** Workflow of Dijkstra algorithm.

Input: gene interaction network (V, E, W)	
Output: feature of each gene	
1	**For***i* = 1 to N { //N is the number of genes
2	**For***j* = i to N {
3	Initialize :*d*_*i*_ = 0,other *d* is infinity;
4	**While***d*_*i*_ = *d*_*j*_ {
5	**For** m = 1 to M {
6	*d*_*m*_ = *min*⁡[*d*_*m*_,*d*_*i*_ + *W*(*k*,*m*)];
7	}
8	Find the points where *d* is the smallest, mark it as*d*_*i*_;
9	}
10	}
11	}
12	Calculate the features of genes in the shortest path
13	**Return***F*′ = (*F*−*mean*(*F*))/*sd*(*F*);

We extracted the features of genes in the last step. The features should be inputted into SVM (Friedrichs and Igel, 2004) to predict IBD-related genes. The workflow of SVM is shown as Figure 4.

**FIGURE 4:**
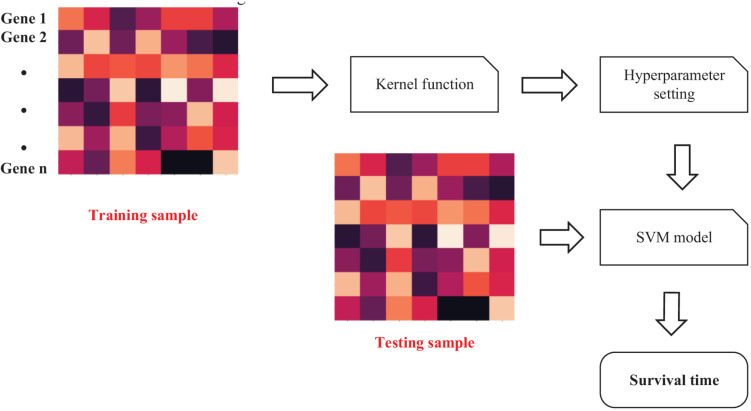
Workflow of Support Vector Machine (SVM)-based prediction method.

Given a training set, *s* = {(*x*_1_,*y*_1_)(*x*_2_,*y*_2_),⋯(*x*_*n*_,*y*_*n*_)},

{*x*_1_…*x*_*n*_} represents the gene expression of patients. {*y*_1_…*y*_*n*_} represents the survival time of patients.

Due to the nonlinear relationship between gene expression and survival time, ϕ(⋅) (kernel function) is needed to map gene expression to high-dimensional feature space.

(3) $$\varphi \,\left(x\right)=\left(\phi \,\left(x_{1}\right),\phi \,\left(x_{2}\right)\,\cdots \,\phi \,\left(x_{n}\right)\right)$$

The Gaussian Radial Basis Function (RBF) has radial symmetry, which makes it smooth. At the same time, as a kernel function, the sample space can be transformed to a high-dimensional space with infinite-dimensional feature space, so that it can better deal with nonlinear relationships. The hyperplane of the feature space can arbitrarily divide the area of the input sample, which can avoid the situation of excessive concentration of training samples.

(4) $$K\,\left(x,y\right)=\exp\left(-{\left(x-y\right)}^{2}\,/\,2\,\sigma^{2}\right)$$

σ is kernel width parameter.

Then perform linear regression, the regression function is:

(5) $$f\,\left(x\right)=w^{T}\cdot \phi \,\left(x\right)+b$$

*w*^*T*^ is weight vector and b is bias. This functional formula could describe relationship between gene expression and survival time. For any given gene features, it can be brought into formula (5) to give a corresponding probability that this is an IBD related gene. Therefore, the next step is to obtain *w*^*T*^ and b.

*w*^*T*^ and b could be obtained based on the principle of structural risk minimization. The structural risk is:

(6) $$R=\gamma \cdot R_{e\,m\,p}+\frac{1}{2}\,{||w||}^{2}$$

Where γ is regularization parameter, *R*_*emp*_ represents the empirical risk function. In our method, the square of the training error was used as the empirical risk function.

(7) $$R_{e\,m\,p}=\sum_{i}^{n}\varepsilon_{i}^{2}$$

To minimize *R*, Lagrange Multiplier Method was introduced.

(8) $$L\,\left(w,b,\varepsilon_{i},a\right)=\gamma \cdot \sum_{i}^{n}\varepsilon_{i}^{2}+\frac{1}{2}\,{||w||}^{2}-\sum_{i=1}^{n}\left(a_{i}\cdot \left(w^{T}\cdot \phi \,\left(x\right)+b-y_{i}\right)\right)$$

Following the Optimal conditions,

(9) $$\left\{ \begin{array}{c} \frac{\partial L}{\partial w}=0\to w=\sum_{i=1}^{n}a_{i}\cdot \phi \,\left(x_{i}\right)\\ \frac{\partial L}{\partial b}=0\to \sum_{i=1}^{n}a_{i}=0\\ \frac{\partial L}{\partial \varepsilon_{i}}=0\to a_{i}=2\,\gamma \,\varepsilon_{i}\\ \frac{\partial L}{\partial a_{i}}=0\to y_{i}=w^{T}\cdot \phi \,\left(x_{i}\right)+b+\varepsilon_{i} \end{array}\right.$$

Therefore, the final SVM model could be represented as:

(10) $$y_{i}=\sum_{j=1}^{n}\left(a_{j}\cdot \left\langle \phi \,\left(x_{j}\right),\phi \,\left(x_{i}\right)\right\rangle \right)+b+\frac{1}{2\,\gamma }\,a_{i}$$

## Results

### Performance of SVM-Based Method in Predicting Survival Time

**FIGURE 5:**
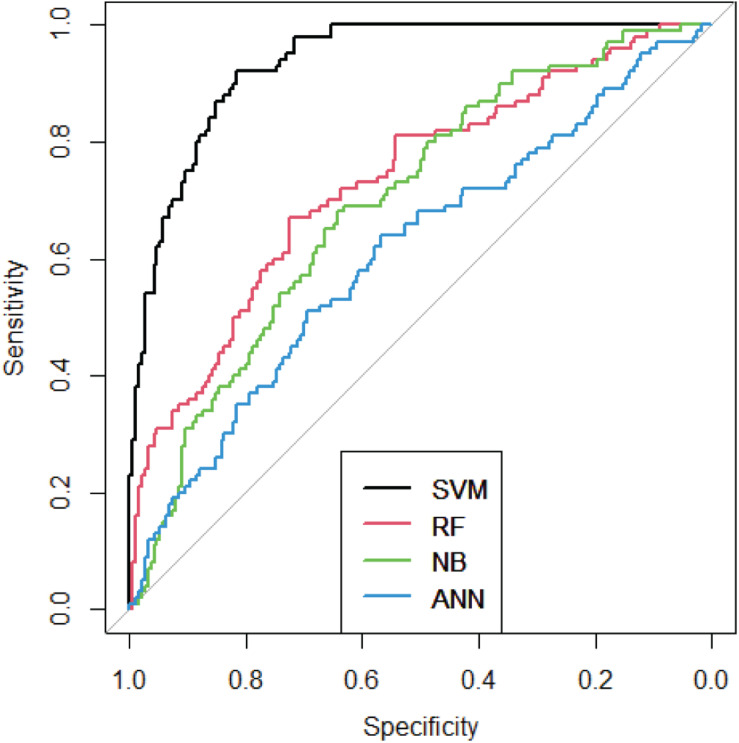
ROC curves of four methods.

To verify the effectiveness of our method, 10-fold cross validation was used. We randomly classified 5,928 genes into 10 groups. Then we used 9 of 10 groups to build the SVM model and the rest 1 group was used as the testing set. The process could be repeated 10 times. Therefore, each group could be trained by 9 times and tested by once. We compared our method with several traditional methods, such as Artificial Neural Network (ANN) (Plumb et al., 2005), Random Forest (RF) (Archer and Kimes, 2008), Naïve Bayes (NB) (Archer and Kimes, 2008). The AUC curves were shown as Figure 5.

As we can see from Figure 5, SVM performed best among these methods. The AUC of SVM is 0.93, which means the IBD-related genes could be precisely predicted.

**FIGURE 6:**
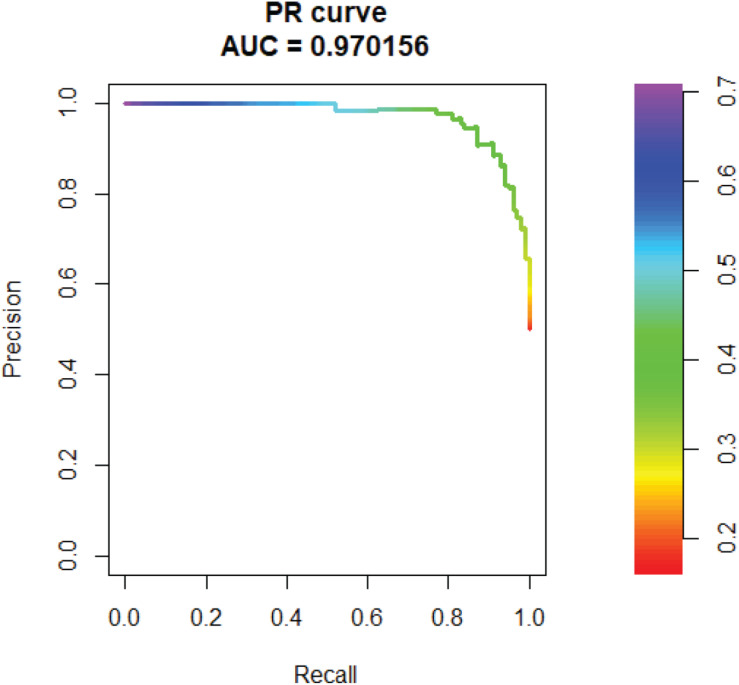
PR curves of Support Vector Machine (SVM).

In addition, we tested the AUPR of SVM. The AUPR is 0.97, which means the false positive rate is low (Figure 6).

### Identify IBD-Related Genes

Since we have proven the effectiveness of SVM in section “Performance of SVM-Based Method in Predicting Survival Time,” we could use this method to identify IBD-related genes in this section.

We totally obtained 1,577 genes related to IBD according to DisGeNET. All these 1,577 genes were used as positive samples to build SVM model. Then, to keep the balance of positive and negative sample sizes, we randomly selected 1,577 genes from 5,928 candidate genes as the negative samples. Finally, the final SVM model could be built. We test whether the rest 4,351 genes are related to IBD by this final SVM model and found that 231 novel genes are related to IBD.

### Case Study

In IBDs, intestinal inflammation has been reported to be accelerated by dysfunction in the epithelial paracellular barrier formed by tight junctions (TJs). Some of the intestinal claudin-family proteins, which form the paracellular barrier, show aberrant expression levels and localizations in IBDs. The intestine-specific Cldn7 deficiency caused colonic inflammation, even though TJ structures were still present due to other claudins according to the study of Tanaka et al. (2015). The paracellular flux (pFlux), determined by measuring the paracellular permeability across the colon epithelium, was enhanced by the Cldn7 deficiency for the small organic solute Lucifer Yellow (457 Da), but not for the larger organic solute FITC-Dextran (4,400 Da). LPA promotes platelet aggregation and induces cellular tension and cell surface fibronectin assembly (Olorundare et al., 2001), which are also important events in wound repair suggesting an important role of LPA in inflammatory disorders. This was confirmed by our group when we demonstrated, that LPA not only promotes epithelial wound healing *in vitro* by a TGF-β-independent pathway, but also ameliorates experimental colitis in an experimental model of colitis in rats (Sturm et al., 2002). LSAMP has been reported to be associated with UC, which is similar to IBD (Brant et al., 2017). A recent study has also found a single-nucleotide polymorphism at rs309 in the *MDM2* gene was associated with UC. They found that people with the GG phenotype of the *MDM2* gene were more prone to UC than those with the TT genotype (Doulabi et al., 2020). Although IBD is not considered as an autoimmune disease, it may trigger autoimmunity caused by the increased antigenic load and mucosal immune activation. Several genetic alterations are shared between IBD and autoimmune diseases. IBD-related inflammation consists of a variety of abnormalities in humoral and cell-mediated immunity, and a generalized enhanced reactivity against intestinal bacteria. The IFN signature in autoimmune diseases represents a useful tool as a biomarker of disease progression and treatment efficiency. In a recent study, they found that OAS1 is a significant IFN response gene which could serve as an early predictor of disease activity and progression, as well as a supplementary therapeutic target (Andreou et al., 2020).

## Conclusion

Increasing evidence have shown the relationship between genetics factors and IBD. Identifying IBD-related genes can further reveal the pathogenesis of IBD and provide important support for clinical diagnosis and treatment. A large number of studies have discovered IBD-related genes by biological experiments. However, few genes have been identified.

Since computational methods have shown strong reliability in identifying diseases-related molecular, in this paper, we proposed a SVM-based method for identifying IBD-related genes. A gene network was constructed based on gene interactions and candidate genes. Candidate genes were selected according to the known related genes of diseases similar to IBD. To extract features of genes, we applied Dijkstra algorithm to extract the network topology characteristics as the features of genes. Finally, a SVM-based model was built to identify IBD-related genes.

To verify the effectiveness of our method, we compared SVM with RF, NB, and ANN. SVM showed highest AUC and AUPR among the 10-fold cross validation. After confirming the accuracy of SVM, we built a model using all the data and obtained 231 new genes related to IBD. In order to verify the accuracy of the results, we conducted a case study. Previous studies have shown that five of our newly discovered genes are related to IBD.

Overall, we proposed a novel method of identifying IBD-related genes in large-scale. This method could be also extended into other diseases to help researchers find more diseases-related genes.

## Data Availability Statement

The datasets presented in this study can be found in online repositories. The names of the repository/repositories and accession number(s) can be found in the article/supplementary material.

## Ethics Statement

Ethical review and approval was not required for the study on human participants in accordance with the local legislation and institutional requirements. Written informed consent for participation was not required for this study in accordance with the national legislation and the institutional requirements.

## Author Contributions

CC provided the idea for the study. LY, YL, and X-DF interpreted the results of the data analyses. All authors contributed to designing the algorithm and network construction, interpretation of the results, read, and approved the final version of the manuscript.

## Conflict of Interest

The authors declare that the research was conducted in the absence of any commercial or financial relationships that could be construed as a potential conflict of interest.

## Publisher’s Note

All claims expressed in this article are solely those of the authors and do not necessarily represent those of their affiliated organizations, or those of the publisher, the editors and the reviewers. Any product that may be evaluated in this article, or claim that may be made by its manufacturer, is not guaranteed or endorsed by the publisher.

## References

[B1] Andreou N.-P. Legaki E. Gazouli M. (2020). Inflammatory bowel disease pathobiology: the role of the interferon signature. *Ann. Gastroenterol.* 33:125. 10.20524/aog.2020.0457 32127733 PMC7049232

[B2] Archer K. J. Kimes R. V. (2008). Empirical characterization of random forest variable importance measures. *Comput. Stat. Data Anal.* 52 2249–2260. 10.1016/j.csda.2007.08.015

[B3] Brant S. R. Okou D. T. Simpson C. L. Cutler D. J. Haritunians T. Bradfield J. P. (2017). Genome-wide association study identifies African-specific susceptibility loci in African Americans with inflammatory bowel disease. *Gastroenterology* 152 206–217. 10.1053/j.gastro.2016.09.032 27693347 PMC5164948

[B4] Cheng L. Li J. Ju P. Peng J. Wang Y. (2014). SemFunSim: a new method for measuring disease similarity by integrating semantic and gene functional association. *PloS One* 9:e99415. 10.1371/journal.pone.0099415 24932637 PMC4059643

[B5] Cheng L. Qi C. Zhuang H. Fu T. Zhang X. (2020). Gutmdisorder: a comprehensive database for dysbiosis of the gut microbiota in disorders and interventions. *Nucleic Acids Res.* 48 D554–D560. 10.1093/nar/gkz843 31584099 PMC6943049

[B6] Danese S. Fiorino G. Peyrin-Biroulet L. (2020). Positioning therapies in ulcerative colitis. *Clin. Gastroenterol. Hepatol.* 18 1280–1290. 10.1016/j.cgh.2020.01.017 31982609

[B7] Doulabi M. S. H. Moghaddam R. G. Salehzadeh A. (2020). Associations between an MDM2 gene polymorphism and ulcerative colitis by ARMS-PCR. *Genomics Inform.* 18:e9. 10.5808/gi.2020.18.1.e9 32224842 PMC7120344

[B8] Franke A. Balschun T. Karlsen T. H. Sventoraityte J. Nikolaus S. Mayr G. (2008). Sequence variants in IL10, ARPC2 and multiple other loci contribute to ulcerative colitis susceptibility. *Nat. Genet.* 40:1319. 10.1038/ng.221 18836448

[B9] Friedrichs F. Igel C. (2004). Evolutionary tuning of multiple SVM parameters. *Neurocomputing* 64 107–117. 10.1016/j.neucom.2004.11.022

[B10] Graham D. B. Xavier R. J. (2020). Pathway paradigms revealed from the genetics of inflammatory bowel disease. *Nature* 578 527–539. 10.1038/s41586-020-2025-2 32103191 PMC7871366

[B11] Hovde Ø Kempski-Monstad I. Småstuen M. C. Solberg I. C. Henriksen M. Jahnsen J. (2014). Mortality and causes of death in Crohn’s disease: results from 20 years of follow-up in the IBSEN study. *Gut* 63 771–775. 10.1136/gutjnl-2013-304766 23744613

[B12] Hwang S. Kim C. Y. Yang S. Kim E. Hart T. Marcotte E. M. (2019). HumanNet v2: human gene networks for disease research. *Nucleic Acids Res.* 47 D573–D580. 10.1093/nar/gky1126 30418591 PMC6323914

[B13] Mayer L. (2010). Evolving paradigms in the pathogenesis of IBD. *J. Gastroenterol.* 45 9–16. 10.1007/s00535-009-0138-3 19960355

[B14] Noto M. Sato H. (2000). “A method for the shortest path search by extended Dijkstra algorithm,” in *Proceedings of the Smc 2000 conference proceedings. 2000 IEEE International Conference on Systems, Man and Cybernetics.’Cybernetics Evolving to Systems, Humans, Organizations, and Their Complex Interactions’(cat. no. 0*, (Nashville, TN: Institute of Electrical and Electronics Engineers), 2316–2320.

[B15] Olén O. Erichsen R. Sachs M. C. Pedersen L. Halfvarson J. Askling J. (2020). Colorectal cancer in Crohn’s disease: a scandinavian population-based cohort study. *Lancet Gastroenterol. Heptol.* 5 475–484. 10.1016/S2468-1253(20)30005-4 32066530

[B16] Olorundare O. E. Peyruchaud O. Albrecht R. M. Mosher D. F. (2001). Assembly of a fibronectin matrix by adherent platelets stimulated by lysophosphatidic acid and other agonists. blood. *J. Am. Soc. Hematol.* 98 117–124. 10.1182/blood.v98.1.117 11418470

[B17] Piñero J. Bravo À Queralt-Rosinach N. Gutiérrez-Sacristán A. Deu-Pons J. Centeno E. (2016). DisGeNET: a comprehensive platform integrating information on human disease-associated genes and variants. *Nucleic Acids Res.* 45 D833–D839. 10.1093/nar/gkw943 27924018 PMC5210640

[B18] Plumb A. P. Rowe R. C. York P. Brown M. (2005). Optimisation of the predictive ability of artificial neural network (ANN) models: a comparison of three ANN programs and four classes of training algorithm. *Eur. J. Pharm. Sci.* 25 395–405. 10.1016/j.ejps.2005.04.010 15893460

[B19] Poulopoulos M. Cortes E. Vonsattel J.-P. G. Fahn S. Waters C. Cote L. J. (2012). Clinical and pathological characteristics of LRRK2 G2019S patients with PD. *J. Mol. Neurosci.* 47 139–143. 10.1007/s12031-011-9696-y 22194196 PMC3335886

[B20] Roda G. Ng S. C. Kotze P. G. Argollo M. Panaccione R. Spinelli A. (2020). Crohn’s disease. *Nat. Rev. Dis. Primers* 6 1–19. 10.1038/s41572-020-0156-2 32433463

[B21] Sturm A. Zeeh J. Sudermann T. Rath H. Gerken G. Dignass A. U. (2002). Lisofylline and lysophospholipids ameliorate experimental colitis in rats. *Digestion* 66 23–29. 10.1159/000064418 12379812

[B22] Tanaka H. Takechi M. Kiyonari H. Shioi G. Tamura A. Tsukita S. (2015). Intestinal deletion of claudin-7 enhances paracellular organic solute flux and initiates colonic inflammation in mice. *Gut* 64 1529–1538. 10.1136/gutjnl-2014-308419 25691495

[B23] Tianyi Z. Yang H. Valsdottir L. R. Tianyi Z. Jiajie P. (2020). Identifying drug–target interactions based on graph convolutional network and deep neural network. *Brief. Bioinform.* 22 2141–2150. 10.1093/bib/bbaa044 32367110

[B24] Van der Sluis M. De Koning B. A. De Bruijn A. C. Velcich A. Meijerink J. P. Van Goudoever J. B. (2006). Muc2-deficient mice spontaneously develop colitis, indicating that MUC2 is critical for colonic protection. *Gastroenterology* 131 117–129. 10.1053/j.gastro.2006.04.020 16831596

[B25] Zhang S. He X. Zhang R. Deng W. (2021). LncR2metasta: a manually curated database for experimentally supported lncRNAs during various cancer metastatic events. *Brief. Bioinform.* 22:bbaa178. 10.1093/bib/bbaa178 32766766

[B26] Zhao T. Hu Y. Cheng L. (2020a). Deep-DRM: a computational method for identifying disease-related metabolites based on graph deep learning approaches. *Brief. Bioinform.* 13:bbaa212. 10.1093/bib/bbaa212 33048110

[B27] Zhao T. Hu Y. Peng J. Cheng L. (2020b). DeepLGP: a novel deep learning method for prioritizing lncRNA target genes. *Bioinformatics* 36 4466–4472. 10.1093/bioinformatics/btaa428 32467970

[B28] Zhao T. Hu Y. Zang T. Cheng L. (2020c). MRTFB regulates the expression of NOMO1 in colon. *Proc. Natl. Acad. Sci. U.S.A.* 117 7568–7569. 10.1073/pnas.2000499117 32184333 PMC7148557

[B29] Zhao T. Hu Y. Zang T. Wang Y. (2019). Integrate GWAS, eQTL, and mQTL data to identify alzheimer’s disease-related genes. *Front. Genet.* 10:1021. 10.3389/fgene.2019.01021 31708967 PMC6824203

[B30] Zhao T. Lyu S. Lu G. Juan L. Zeng X. Wei Z. (2021). SC2disease: a manually curated database of single-cell transcriptome for human diseases. *Nucleic Acids Res.* 49 D1413–D1419. 10.1093/nar/gkaa838 33010177 PMC7778914

